# Investigating potent cardioprotective compounds as ACE inhibitors in *Saraca asoca*

**DOI:** 10.1016/j.toxrep.2024.101731

**Published:** 2024-09-10

**Authors:** Sonal Prasad, Kaiser Younis, Owais Yousuf

**Affiliations:** a Faculty of BioSciences, IBST, Shri Ramswaroop Memorial University, Lucknow, Deva road, Barabanki-22500, India; b Department of Food Technology, Islamic University of Science and Technology, Kashmir, India

**Keywords:** *Saraca asoca*, Natural bioactive compounds, Cardio-protective, ACE inhibitor

## Abstract

*Saraca asoca* is a traditional medicinal plant whose all plant parts are exceptionally effective in terms of antimicrobial, anti-inflammatory, antioxidant, anti-carcinogenic, free radical scavenging,anti-arthritic, and hypolipidemic properties. As cardio-vascular problems occur for many reasons, antioxidants with free radical scavenging properties of plants and herbs are highly effective in treating cardio-related disorders. Though *Saraca asoca* has been preferred as a tonic and medicinal supplement for women's health, because of the huge variety of bioactive compounds, *Saraca asoca* needs to be explored for its cardio-protective properties. This review aims to summarize the *in vivo* and *in vitro* studies done on *Saraca asoca* along with the exploration of bioactive compounds in various parts of the plant which will display its cardio-protective potential with its rich bioactive compounds as ACE inhibitors. All relevant information on *Saraca asoca* in treating and preventing cardio-related disorders has been collected from electronic databases including PubMed, Google Scholar, Web of Science, and Science Direct. Various parts of *Saraca asoca* were studied to assess its pharmacological and cardioprotective properties. The bioactive compounds of *Saraca asoca* have been assessed to explore its role as anti-hypertensive, antioxidant, ACE inhibitors, and cardio-protective with the help of *in-vivo*, *in-vitro* studies and other research studies. This thorough review focuses on the potent natural bioactive compounds in various parts of *Saraca asoca* exhibiting its potential as a cardioprotective agent while incorporating historical, chemical, and therapeutic views.

## Highlights

• Potent natural bioactive compounds of Saraca asoca explored for the potential cardio-protective agent.
• Studies on Saraca asoca were assessed demonstrating its cardio-protective potential and acting as ACE inhibitors
• Highlighted the natural bioactive compounds in various parts of Saraca asoca as a potential cardioprotective agent.

## Graphical abstract

**Figure:**
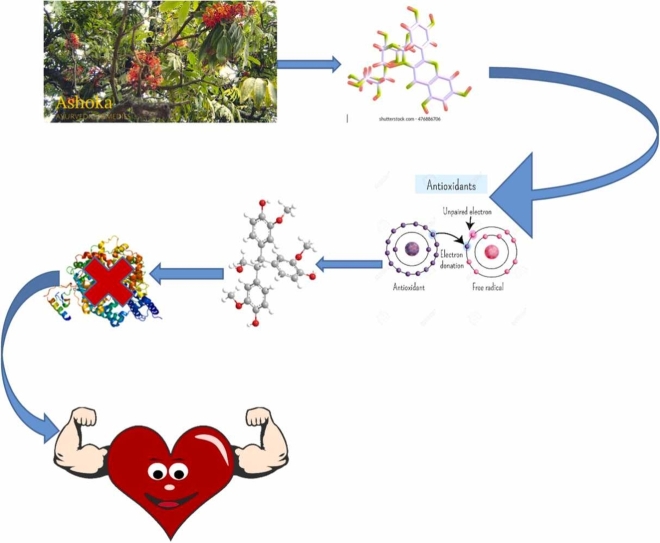

## 1 Introduction

Cardiovascular disease (CVD) remains one of the major public health problems responsible for morbidity, mortality, and many deaths worldwide [1]. Many factors in accelerate the risk of cardiovascular diseases are hypertension, physical inactivity, stress, smoking, faulty food habits, improper lifestyle, environmental pollutants, toxic heavy metals, reactive oxygen species, etc. Apart from aggravating other serious cardiovascular problems such as atherosclerosis, coronary artery disease, and stroke, hypertension plays a key role in damaging the kidneys well [2]. As per many reports and research studies, reactive oxygen species and free radicals such as superoxide anion, hydroxyl radical, and hydrogen peroxide, are the prominent cause of increasing the risk of hypertension [3]. So, to mitigate the risk of cardiovascular diseases, it is suggested by doctors, scientists, and nutritionists to increase the intake of strong antioxidants and essential nutrients by consuming herbs and herbal-related products in the daily diet regularly. As many plants and herbs contain essential nutrients along with natural bioactive compounds, their increased consumption is strongly recommended due to their free radical scavenging, anti-inflammatory, antioxidant, and other medicinal properties in protecting the body against the toxic effects of free radicals and reactive oxygen species. Apart from this, plants and herbs have also the ability to act as heavy metal chelators proving them to be effective hyperaccumulators [4]. Thus, the prime focus in treating and preventing cardiovascular-related problems could be the use of natural bioactive compounds extracted from herbs and plants acting as cardioprotective agents.

In this context, among many herbal plants, *Saraca asoca*, one of the traditional plants used as a medicine in Ayurveda needs to be explored for its effective cardio-protective properties. It is one of the oldest and sacred trees in Indian religion. It is found almost all over the country but it is mainly predominantly present in the northern areas of the country along with Eastern Bengal, South India, Aracan, and Tenasserium. All parts of the Saraca asoca plant are highly effective from the health benefits perspective. In the classical Ayurvedic treatise of Charaka Samhita (1000 BC), *Saraca asoca* is an analgesic and astringent and a tonic for women's health [5], [6]. The taxonomic classification of Saraca asoca is of *Kingdom: Plantae; Divison: Magnoliophyta; Class: Magnoliopsida; Order: Fabales; Family: Fabaceae; Genus:* Saraca and *Species:* asoca[7], [8].

**Fig. 1:**
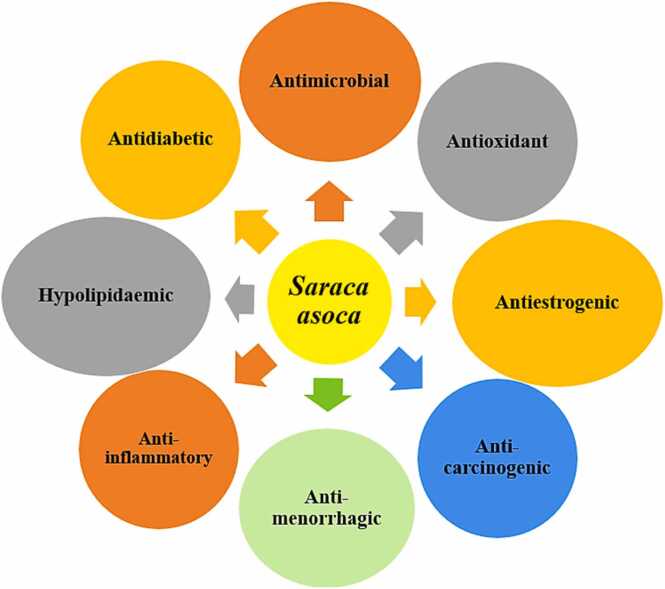
Medicinal and health properties of *Saraca asoca*.

*Saraca asoca* is well known for its antimicrobial, larvicidal, antidiabetic, anticarcinogenic, antioxidant, antimennorhagic, oxytocic, anti-estrogenic, and anti-inflammatory properties thus protecting the human body against the adverse effects of free radicals and other toxic heavy metals thus showing its rich potential benefits from the medicinal perspective (Fig. 1) [9], [10], [11]. Usually, *Saraca asoca* has been well-known for treating women's health-related disorders many centuries ago, such as menorrhagia, leucorrhoea, dysfunctional uterine bleeding, hemorrhoids, etc.[12]. However, due to the presence of many natural bioactive compounds in every part of the plant, it is considered a highly valuable endangered plant whose medicinal benefits need to be explored in treating other disorders as well [13], [14]. As per many *in-vivo* and *in-vitro* studies, the natural compounds present in this plant have been found to act as hyperaccumulators and health protective. So, it could be an innovative study to explore the role of *saraca asoca* as a cardio-protective herb and use it in the development of medicinal drugs and nutraceuticals as well. It is well known that superoxide anion radical is a highly toxic particulate matter pollutant and is a major source of reactive oxygen species which is responsible for the development of dangerous hydroxyl radicals contributing to oxidative stress [3]. A research study on the methanol leaf and bark extract of *Saraca asoca* has shown that it has the maximum superoxide radical scavenging activity [15].

Numerous studies support the phytochemical, antibacterial, and antioxidant activity of the whole plant extract of Saraca asoca, making it valuable for the ayurvedic and pharmaceutical industries' production of nutraceuticals. This emphasizes the significance of further investigation into the plant's nutritional and medicinal qualities, as they are increasingly important to healthcare. Due to its ability to heal problems relating to women, Saraca asoca has been the subject of numerous studies on the treatment of gynecological disorders and is a good female tonic. However, very little research has been done on this valuable plant's antihypertensive cardioprotective qualities. Consequently, research into probable naturally occurring bioactive compounds from Saraca asoca with the ability to block ACE could prove helpful for cardioprotection.

## 2 Phytochemical study

Many studies have proved that all parts of *Saraca asoca* are highly beneficial in terms of medicinal properties. Many potent natural bioactive compounds are present in this plant which have proven to be very beneficial in terms of antimicrobial, antioxidant, oxytocic, anti-estrogenic, antidiabetic, anticarcinogenic**,** antimennorhagic, and anti-inflammatory properties. So, most parts of this plant have been reviewed for its potential cardio-protective properties.

### 2.1 Bark

**Fig. 2:**
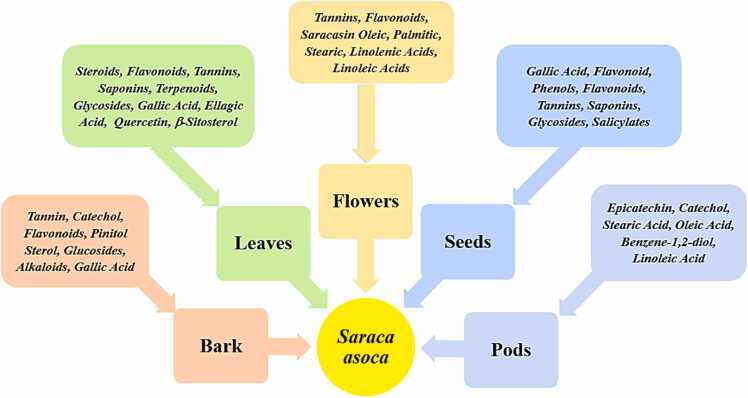
Natural bioactive compounds in different parts of Saraca asoca with cardio-protective properties.

Bark of *Saraca asoca* contains many effective bioactive compounds such as tannin, catechol, flavonoids, sterol, glucosides, and alkaloids including other organic calcium compounds [16], which are very potent in acting as a tonic for females in treating gynecological disorders such as menstrual disorders, leucorrhea[17]. The effective potent antioxidants and bioactive compounds *such as* catechin, flavonoids, lignin glycosides, beta-sitosterol, and its glucosidic form, polyphenolics along with gallic acid and methanol extracts are present in leaves, bark, and flower *Saraca asoca exhibiting its strong* antioxidant, antidiabetic and hypolipidemic properties (Fig. 2)[18], [19], [20], [21], [22], [23], [24], [25], [26], [27], [28].

In a study, the *Saraca asoca* flora and leaves have exhibited rich flavonoid properties lowering LDL (low-density lipoprotein) oxidation in the blood which is one of the main reasons for cardiovascular diseases and inhibiting the activity of α-glucosidase and α- amylase enzymes as well which are responsible in accelerating type-2 diabetes. [19], [26], [29]. A study done on albino rats and mice has shown that S. asoca extracts are effective in decreasing lipid and cholesterol levels in blood proving it to be cardioprotective. [19], [29].

### 2.2 Leaves

Leaves of *Saraca asoca* contain bioactive compounds such as alkaloids, steroids, flavonoids, tannins, saponins, terpenoids, glycosides, and polyphenolics such as gallic acid and ellagic acid along with flavonoids. Flavonoids such as quercetin, β-sitosterol, ceryl alcohol, and glucosides such as quercetin-3-O-α-rhamnoside and kaemferol-3-O-α-Lrhamnoside have proven to be highly effective in protecting the human heart (Fig. 2)[30], [31], [32]. In a study, the leaves, bark, and roots of *Saraca asoca containing* ethanolic and methanolic extracts have shown anti-inflammatory properties by preventing the binding of transcription factors such as NF-κB, AP-1, GATA-1, etc. to their target DNA sequences thus decreasing the proinflammatory cytokines [18].

### 2.3 Seeds and pods

Pods of *Saraca asoca* contain stearic acids with sterols namely epicatechol catechol [33]. Apart from flavonoids, phenols, flavonoids, tannins, saponins, glycosides, and salicylates, these compounds are also found in the seeds [31]. Seeds and pods of *Saraca asoca* contain bioactive compounds Oleic acid and Linoleic acid which are anti-inflammatory and a proven cardioprotective for a healthy human heart (Fig.2) [34].

### 2.4 Flowers

A study was done on S. asoca flower extract's anti-cancer potential against lung cancer cell lines. The lung cell line (A549) was used in a cell viability assay to test the cytotoxic effect of S. asoca flower extract. For a full day, various dosages of ethanolic S. asoca flower extract (20–120 µg/mL) were administered. Furthermore, inverted microscopy was used to analyze the morphological alterations. Using DAPI labeling, the nuclear morphology of lung cancer treated with an ethanolic extract from S. asoca flowers was examined. The study demonstrates that treating lung cancer cells with an ethanolic extract of S. asoca flowers reduces cell proliferation and triggers apoptosis [35].

## 3 Cardioprotective bioactive compounds in *Saraca asoca*

Free radical generation is responsible for many disorders including atherosclerosis, ischemia, arthritis, and coronary heart diseases. Therefore, strong antioxidants are endorsed for mitigating the risk of cardiovascular diseases. Bioactive compounds such as Beta-sterol, isoflavones, flavonols, like catechin, Epicatechin, Gallocatechin, Epigallocatechin, rutin, and quercetin, fatty acids like oleic, stearic, linolenic, linoleic acids,9,12-Octadecadienoic acidhave proven themselves as a protective guard for the human heart. So, in this study, the bioactive compounds in the context of *Saraca asoca* as a cardioprotective have been assessed with the help of many investigational *in-vivo* and *in-vitro* studies. Table 1

**Table 1:** Bioactive compounds from different plant parts of *Saraca asoca* a potent antioxidant and cardio-protective.

**Bioactive compound**	**Class of compound**	**Plant Part**	**Biological function**	**Reference**
Gallic acid	Phenolic acid	Bark, flowers, leaves	Hypolipidemic, Antioxidant	Abdelmoaty, M. A. et al. [36]
Ellagic acid	Phenolic acid	Bark	Antioxidant	Saha, J. et al. [37]
Quercetin	Phenolic acid	leaves	Antioxidant	Angeloni C. et al. [38]
Epicatechin	Flavonol	Seed, pod, bark	Anti-Inflammatory, Antimicrobial	Ketkar PM et al. [39]
Gallocatechin	Flavonol	Bark	Anti-Inflammatory, Antimicrobial	Ahmad, F. et al. [40]
Epigallocatechin	Flavonol	Bark	Anti-Inflammatory, Antimicrobial	Ahmad, F. et al. [40]
β-Sitosterol	Sterol	Bark, leaves, and stem	Hypolipidemic, Antioxidant, Antidiabetic	Hegde, S. et al.; Mishra, A et al. [8], [41]
β-Sitosterol glucoside	Steroidal glycoside	Bark, leaves	Hypolipidemic, Antioxidant, Antidiabetic	Hegde, S. et al.; Mishra, A et al. [8], [41]
Stearic acid	Fatty acid	Seed, pod	Anti-Inflammatory, Cardioprotective	Kalakotla, S. et al. [34]
Oleic acid	Fatty acid	Seed, pod	Anti-Inflammatory, Cardioprotective	Kalakotla, S. et al. [34]
Linoleic acid	Fatty acid	Seed, pod	Anti-Inflammatory, Cardioprotective	Kalakotla, S. et al. [34]
Benzene−1,2-diol	Catechin	Seed, pod, bark	Anti-Inflammatory, Antimicrobial	Shirolkar, A. et al. [42]

### 3.1 β-Sitosterol

**Fig. 3:**
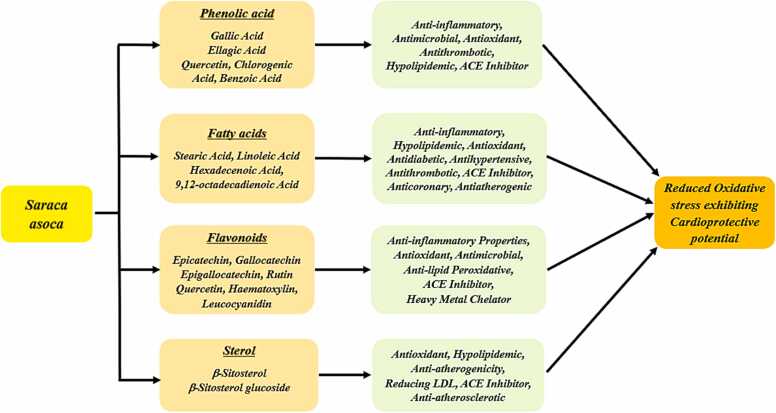
Health benefits of various bioactive compounds predominantly present in *Saraca asoca* exhibiting Cardio-protective properties.

β-sitosterol is a naturally occurring compound that is found in many herbs and plants having many antioxidant, anticancer, anti-inflammatory, and immunomodulatory properties (Fig. 2). Beta-sterol is an effective bioactive compound present in the bark, stem, and leaves of *Saraca asoca.* The bark, leaves, and stem of *Saraca contain* bioactive compounds β-Sitosterol which are rich in antioxidant and hypolipidemic properties with a strong effect on lowering the bad cholesterol (LDL level) and maintaining a healthy heart [8], [21], [22], [23], [41]. The role of beta-sitosterol as an ACE inhibitor was studied and it was assessed that it plays a significant role in inhibiting the conversion of angiotensin I to angiotensin II thus controlling the systolic blood pressure (Fig. 3) [43]. Table 2

**Table 2:** Bioactive compounds interact *with asoca* exhibitingCardioprotectiveproperties.

**Bioactive compounds of*****Saraca asoca***	**Medicinal properties and probable use**	**Reference**
**β-Sitosterol-**	Antioxidant, and hypolipidemic properties	Plat, J. et al. 2019; Pradhan, P. et al. 2010; Mishra, A. et al. 2010; Khan, H. et al. 2020 [30], [41], [44], [43]
**Fatty acids-**	Anti-inflammatory properties, increase high-density lipoprotein (ie, good cholesterol) levels.	Saha, J. et al. 2013; Pourmorad, F.et al.2006 [37], [45]
**Galloylated catechins**	Anti-inflammatory properties, free radical scavenger, anti-carcinogens	Kawai Y et al.2008 [46]
**Rutin and Quercetin-**	Strong antioxidant and anti-inflammatory properties, free radical scavenger	Angeloni C. et al. 2007; Jain, A. et al. 2013; Mohan, Ch. et al. 2016 [14], [19], [38]
**Hexadecanoic acid or Palmatic acid**	Antioxidant, hypocholesterolemic, anti-inflammatory, antibacterial activities	Bangajavalli 2019 [47]
**9,12-Octadecadienoic acid (*Z*,*Z*)or Linoleic acid**	Hypocholesterolemic, antibacterial, hepato-protective, Antihistaminic, antiarthritic, anti-coronary, Anti-inflammatory properties, free radical scavenger	Bangajavalli 2019 [47]

### 3.2 Fatty acids

Many fatty acids including stearic, palmitic, oleic, linolenic, and linoleic acids have effective cardioprotective properties that protect the cardiac tissue against the detrimental effect of free radicals and reactive oxygen species [37]. In a study, it has been proven that the flowers of *Saraca asoca have* tannins, flavonoids, and saracasin along with fatty acids such as oleic, palmitic, stearic, linolenic, and linoleic acids which have shown strong cardioprotective properties by protecting the cardiac tissue from the infiltration of inflammatory cells (Fig. 3) [37]. Usually, the antioxidant activity is associated with the biological actions of the two bioactive compounds flavonoids and polyphenols [48]. As flavonols and phenolic compounds have anti-inflammatory properties and are predominantly present in *Saraca asoca*, they can inhibit the activity of free radical scavenging of hydrolytic and oxidative enzymes, thus showing their strong antioxidant potential as a cardiotonic (Fig. 3) [45].

### 3.3 Flavonols

The flavonols such as Leucocyanidin, Epicatechin, Gallocatechin, Epigallocatechin, Haematoxylin, Quercetin-3-O-P-D-glucoside present in seeds, pods, bark, flowers, leaves of *Saraca asoca* possess the anti-inflammatory, antimicrobial, antioxidant properties showing a strong free radical scavenging activity and also proving it to a cardioprotective tonic (Fig. 3). [40], [49], [42], [50]. In a study of the bark of *S. asoca*, epicatechin was found to be higher in comparison than gallic acid in all seasons with the application of the Reverse Phase High-Performance Liquid Chromatography-Diode Array Detector (RP-HPLC-DAD) method [39].

### 3.4 Rutin and quercetin

In a study, rats with experimental streptozotocin (STZ)-induced diabetes were used to evaluate the potential effects of QE on antioxidant enzymes and blood glucose. Normal animal plasma glucose levels were unaffected by quercetin, but diabetes caused by a single intraperitoneal injection of streptozocin-treated rats may be avoided with its pretreatment. In the STZ-induced diabetes group, antioxidant enzyme activity dramatically dropped. The activities of antioxidant enzymes were dramatically boosted by QE treatment. It could be taken that the flavonoid quercetin, which has antioxidant qualities, is helping to prevent diabetes [36]. In another in-vivo study done on rats, the effects of flavonoids rutin and quercetin were investigated and it was observed that both flavonoids were highly effective in scavenging the free radicals and inhibiting ferrous ion-dependent lipid peroxidation of lecithin liposomes and In NADPH In- In and In CCl4-dependent Inlipid In peroxidation in In rat In liver microsomes showing its strong antioxidant and metal chelating properties [38].

Rutin (RUT) is a citrus flavonoid glycoside and is widely used in medicine for the maintenance of capillary integrity. Quercetin (QCT) is 3,5,7,3′,4′- pentahydroxyflavone and is a potent compound in preventing cardio-vascular disorders (Fig. 3) [51]. Swiss Albino mice were used in a research study for the assessment of radiation-induced sickness with the anti-oxidative properties of RUT & QCT where elevation in the antioxidant status, anti-lipid peroxidative potential were demonstrated and potential of RUT and QCT were explored [52]. In an *in vitro* study, Galloylated catechins have higher ACE inhibitory activity thus proving their strong cardioprotective properties [53]. Recently, in an *in vitro* study, a bioactive compound pinitol has been isolated from the bark of *Saraca asoca* which is better known for its anti-inflammatory activities. Due to its numerous biological properties, such as its hypoglycemic and anti-inflammatory effects, pinitol is a widely recognized bioactive substance.A method for isolating a comparatively high concentration of (+)-pinitol from S. asoca bark has been devised, and its anti-inflammatory and anti-TNF-α properties against carrageenan-induced edema have been validated in vitro. During investigations into potential agonistic activity, (+)-pinitol was found to reduce β-lactam antibiotic dosages by up to eight times[54]. A study on the methanol bark extract of *Saraca asoca* showed maximum DPPH, & radical scavenging activity indicating strong antioxidant & anti-inflammatory properties [14].

### 3.5 Hexadecanoic acid

In an investigational study to analyze the bioactive compounds of Saraca asoca bark, GC-MS analysis of ethanolic extract of Saraca asoca bark showed that n-Hexadecanoic acid compound found in *Saraca asoca* possess antioxidant, hypocholesterolemic, antibacterial activities, anti-inflammatory, and 9,12-Octadecadienoic acid (*Z*,*Z*)- compound have hypo-cholesterolemic, anti-bacterial, hepato protective, antihistaminic, anti-arthritic, anti-coronary, anti-inflammatory properties thus inter-relating its positive effect on heart (Fig. 3) [47]. 9,12-Octadecadienoic acid (*Z*,*Z*)- has hypocholesterolemic, antibacterial, hepatoprotective, Antihistaminic, antiarthritic, anti-coronary, and Anti-inflammatory properties (Fig. 3) [47].

### 3.6 Galloylated catechins

Studies have shown that green tea catechins, especially 3-galloylated catechins have been very potent in inhibiting inflammation-derived DNA damage and thus tumor-causing carcinogens [46]. In an *in vitro* study, Galloylated catechins have higher ACE inhibitory activity thus proving their strong cardioprotective properties [53]. This compound makes *Saraca asoca* to be explored for its ACE inhibitory properties (Fig. 3).

## 4 ACE inhibiting mechanism &cardioprotective Bioactive compounds in *Saraca asoca*

**Fig. 4:**
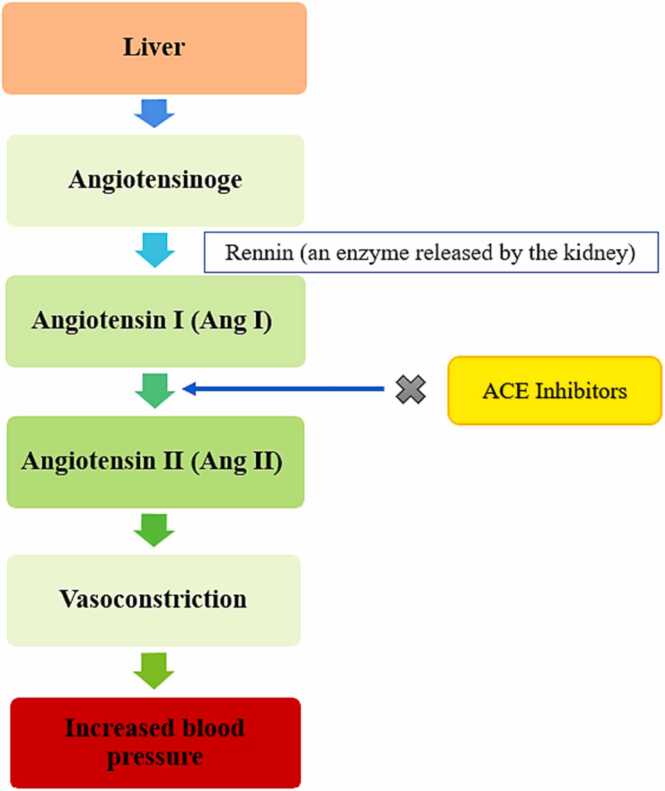
Mechanism of angiotensin-converting Enzyme (ACE) in cardiovascular diseases.

ACE (Angiotensin converting enzyme) is one of the most important factors that trigger normal cardio-health by converting angiotensin I to vasoconstrictor angiotensin II and inactivating the bradykinin as well which is an antihypertensive vasodilator [55]. ACE (a Zn^2+^ binding metalloenzyme) is an enzyme found in many crucial body organs including the heart, vascular tissue, adrenal cortex, liver, kidneys, peripheral uterus, and epididymal cells in the endothelial lining of the lungs. Renin is an enzyme released by kidneys that allows blood volume that catalyzes the conversion of angiotensinogen to angiotensin I (Ang I). Angiotensin I (Ang I) is broken down by the Angiotensin Converting Enzyme (ACE)-1 to Angiotensin II (Ang II) which then aggravates the blood pressure by narrowing the blood vessels and blocking the flow of blood. So, high ACE activity in the blood is detrimental to the heart by enhancing the concentration of angiotensin II and increasing the risk of hypertension(Fig. 4). That is why, ACE inhibitors isolated from the plant extracts are considered a healing and curative agent that inhibit the production of angiotensin II preventing hypertensive and other cardio-related problems [56]. The main bioactive constituents of *Saraca asoca* for protecting the cardiac tissue by inhibiting ACE enzyme include flavonoids, flavanols, catechins, anthocyanins, phenolic acids, polyphenols, tannins, resveratrol, polysaccharides, fiber, saponin, sterols have proven to exhibit cardioprotective properties [54].

## 5 Cardio-protective effect of the *Saraca asoca* extract

As per the research, several studies have shown the cardioprotective effect of Saraca asoca. In one of the studies, the investigation focused on the potential cardioprotective effects of an alcoholic bark extract from *Saraca asoca* against cyclophosphamide-induced cardiotoxicity. When cyclophosphamide-induced cardiotoxicity was treated, there was a significant (p<0.05) improvement in the state of cardiac biomarkers, ECG, oxidative enzymes, and lipid profile. The cardioprotective effect of *Saraca indica*, which may be related to antioxidant activity, is supported by histopathological reports, biochemical analyses, and electrocardiograms [57]. One more Research showed the antioxidant and free radical-scavenging qualities of Saraca asoca leaves and bark. DPPH, Superoxide Radical, Hydroxyl Radical, Lipid Peroxidation, ABTS Radical Scavenging Capacity, and Nitric Oxide Scavenging Ability were used to evaluate the antioxidant properties of Saraca asoca methanolic extract. The leaf and bark extract demonstrated impressive IC50 values in a concentration-dependent manner when tested against ARSC, DPPH, Superoxide, Hydroxyl, Lipid Peroxidation, Nitric Oxide, and FRAP. This suggests that it may be a viable antioxidant. The thorough analysis finding of showed that the extract from Saraca asoca leaves and bark had good antioxidant properties and might be used for future medical and nutraceutical applications [58].

Another study done investigating the ACE inhibitory effect of *Angelica decursiva* showed significant potential of the plant as a cardioprotective agent. Using an in vitro ACE assay, researchers extracted 16 coumarins and 2 phenolic compounds from A. decursiva and evaluated their ACE inhibitory efficacy. Surprisingly 11–18 of these naturally occurring coumarins have IC50 values between 4.68 and 20.04 µM. As a result, screening for cardiovascular disorders including hypertension, and creating novel ACE inhibitors from A. decursiva could be effective. The available data further showed that linear furanocoumarins inhibit ACE activity in vitro and that the class and structure of the coumarins affect their activity and method of action against ACE. When creating novel ACE inhibitors based on coumarins, these structure-function correlations could prove valuable [59]. Two potent angiotensin-converting enzyme inhibitors, namely pellucidin A and the newly discovered polyphenol 2,3,5-trimethoxy-9-(12,14,15- trimethoxybenzyl)-1 H-indene, were effectively extracted from *Peperomia pellucida* and used as an antihypertensive medication in an in vitro investigation. Another new substance is called chromene, or (S)-2-methyl-2-(4-methylpent-3-enyl)Propan-2-ylidene, or −6-From the leaves of Peperomia pellucida (Piperaceae), −3,4,6,7-tetrahydropyran (4,3 g) chrome-9 (2 H)-one (1) has been extracted [60]. Both substances have demonstrated strong inhibitory action on ACE, indicating their potential as heart-friendly and cardioprotective agents. Every polyphenol compound found in Peperomia pellucida, including tetrahydrofuran, lignin (1 R,2S,3S,5 R)-3,5-bis(4-hydroxy-3,5- methoxyphenyl), cyclopentane-1,2 diyl) bis-(methylene) diacetate, has the potential to function as a potent ACE inhibitor, according to an in silico molecular docking study on the plant [61].

An ACE-hexapeptide inhibitor (Asp-Glu-Asn-Ser-Lys-Phe) called chebulin was extracted from Terminalia chebula Retz. using pepsin digestion in an exploratory study. Chebulin has been shown in the kinetic study to bind with the ACE inhibitor and inhibit ACE in a noncompetitive way. Chebulin has demonstrated the ability of ACE inhibitors to prevent systolic blood pressure issues and may be utilized in place of ACE inhibitor medications [62]. 32 major bioactive compounds, including various phenolic, sesquiterpene, flavonoid, triazine, and gibberellin compounds, were found in the in vitro study on the GC-MS analysis of Terminalia chebula's acetone extract, indicating its potential as an antioxidant and antibacterial agent [63]. Resveratrol was discovered in the camu-camu(Myrciariadubia McVaugh, Myrtaceae) seed coat as an effective bioactive compound with antihypertensive, and anti-inflammatory properties. It contains polyphenols such as proanthocyanidins, ellagitannins, and ellagic acid derivatives. The presence of stachyurin and casuarinin with some other tannins present in it exhibits its rich antioxidant and anti-inflammatory activities. Phenolic acids and flavonoids along with the free radical scavenging activity of the Camu-camu [Myrciariadubia] and the inhibition of angiotensin-converting enzyme (ACE) were also examined in an in vitro study on these topics [64]. The Echinacea purpurea flower extract showed strong antioxidant qualities in the experimental study. According to the in vitro investigation, the bioactive components caffeic acid and chlorogenic acid showed increased ACE-inhibitory activity and had antihypertensive qualities, indicating that their application could effectively treat cardiac-related issues [65].

It has been observed that a variety of herbs and spices provides food antioxidant benefits. Multiple research studies indicate that the bark of Saraca indica shows antioxidant activity in its ethanolic, hydroalcoholic, and acetone extracts. Saraca indica roxb's in vitro antioxidant activity was investigated by Panchawat and Sisodia. Using an in-vitro DPPH (1, 1, diphenyl-2 picryl hydrazyl) model, De Wilde stems bark investigators discovered a high phenolic content may be the reason for the antioxidant properties of the different extracts [66]. Because of its strong antioxidant action, eating the bark and flowers of S. asoca may be beneficial. Gallic acid and ellagic acid can be found in the flower and bark, respectively. Another possible source of gallic acid could be leaves that contain a considerable amount of it [37]. A study showed different solvents were used to compare the phytochemical and medicinal activity of S. asoca leaf and in vitro callus extracts. Out of all of these, the in vitro callus methanol extracts had the highest concentration of flavonoids (655.33 ± 42.51 mg QE/g extract) and phenolics (984.20 ± 23.14 mg GAE/g extract). Better scavenging (IC50 38.79 ± 2.35 mg/mL) and chelating properties (98.79 ± 3.35 mg EDTA E/g extract) were also demonstrated by the 2,2-Diphenyl-1-picrylhydrazy (DPPH) assay. Salmonella typhi (ZOI 14 mm) and Enterococcus faecium (Zone of Inhibition 17 mm) were both significantly inhibited by the callus methanol extract. The callus methanol extract LC-MS analysis revealed eight chemicals, primarily flavonoids like quercetin, naringenin, and epiafzelechin. These results support the theory that S. asoca contains beneficial natural antioxidants that may be used as preventative therapies [67].

An in vitro DPPH model was used to compare the antioxidant activity of Saraca indica and Pterospermumacerifolium with ascorbic acid. Researchers figured out that Pterospermumacerifolium and processed Saraca indica displayed significant antioxidant effects [25]. When compared to the control (normal saline) & standard (Indomethacin) groups, regular treatment up to 21 days of adjuvant-induced arthritic rats with Saraca asoca acetone extract (300 and 500 mg/kg doses) increases RBC and Hb, decreases WBC, ESR, and prostaglandin levels in the blood, and restores body weight. Ankle joint inflammation, paw edema, and urine concentrations of hydroxyproline and glucosamine showed significant (P < 0.05) inhibitory effects, mainly at higher dosages. Its significant nontoxic, antiarthritic, and anti-inflammatory effects were further validated by normal radiographic imaging of the joint and histological study of the joint, liver, stomach, and kidney [19].

## 6 Toxicity and safety of the *Saraca asoca* extract

According to toxicological research, *Saraca indica bark extract is* less harmful to use and may be a complementary and alternative treatment option for breast cancer. *Saraca indica bark extract* does not cause appreciable toxicity, according to research on the effects of repeated doses of the drug in vivo and in vitro cytotoxicity on normal cell lines. Additionally, SIE exhibits strong anticancer and antioxidant properties in vitro. Saraca indica bark extract's anti-breast cancer, antioxidant, and toxicological evaluations are encouraging and suggest that this herbal preparation may find application in complementary and alternative medicine for the treatment of breast cancer. Every hematological parameter is within the normal range and did not significantly differ between the sexes or between dose groups. These findings suggested that, in these experimental conditions, *Saraca indica bark extract has* no negative effects on blood. According to the current toxicological research, *Saraca indica bark extract* is a safer alternative therapeutic agent because it does not exhibit any toxicological symptoms [28]. Many studies have documented the diverse adverse effects of herbal plants, even though medicinal plants employed as therapeutic agents are thought to be safe for human ingestion. Medicinal plant usage for health benefits is not consumed without a doctor's advice and proper training. Even though people have been utilizing medicinal herbs for centuries, these plants still need to have their safety evaluated [25]. In addition to compensating for the toxicity and increased bioavailability of active compounds, extracts from medicinal plants exhibit a synergistic effect by acting at different or concurrent nodes within a cancer signaling network, which increases the therapeutic potential of the extracts many times over when compared to a single drug-target therapy [68].

## 7 Conclusion

The importance of all parts of *Saraca asoca* has been proven with most of its beneficial properties, especially as a female tonic treating most of the gynecological problems of women. However, many valuable effective bioactive compounds in *Saraca asoca* have demonstrated their anti-inflammatory, antimicrobial, anticarcinogenic, free radical scavenging, and hypolipidemic properties in many previous research studies. Much research has been done on it which has proved it as a tonic for women's health as it has been used in treating menstrual disorders, showing anti-breast cancer properties, etc. but exploring it as a cardio-tonic might be an innovative research by evaluating and analyzing its potent bioactive compounds and assessing its ACE inhibiting properties. So, the above-discussed bioactive compounds mentioned in *Saraca asoca* need to be explored in inter-relating its cardioprotective properties by investigating its role as ACE inhibitor by inhibiting the conversion of angiotensin I to angiotensin II thus protecting the heart and minimizing the risk of cardiovascular diseases.

## CRediT authorship contribution statement

**Owais Yousuf:** Writing – review & editing, Supervision. **Kaiser Younis:** Writing – review & editing, Project administration. **sonal prasad:** Writing – original draft, Conceptualization.

## Declaration of Competing Interest

The authors declare that they have no known competing financial interests or personal relationships that could have appeared to influence the work reported in this paper.

## Data Availability

No data was used for the research described in the article.
